# Rapid Identification of SARS-CoV-2 Omicron BA.5 Spike Mutation F486V in Clinical Specimens Using a High-Resolution Melting-Based Assay

**DOI:** 10.3390/v14112401

**Published:** 2022-10-29

**Authors:** Akira Aoki, Hirokazu Adachi, Yoko Mori, Miyabi Ito, Katsuhiko Sato, Masayoshi Kinoshita, Masahiro Kuriki, Kenji Okuda, Toru Sakakibara, Yoshinori Okamoto, Hideto Jinno

**Affiliations:** 1 Faculty of Pharmacy, Meijo University, 150 Yagotoyama, Tempaku-ku, Nagoya 468-8503, Japan; 2 Aichi Prefectural Institute of Public Health, 7-6 Nagare, Tsuji-machi, Kita-ku, Nagoya 462-8576, Japan; 3 Kiyosu Health Center, 129 Haruhi, Furikata, Kiyosu 452-0961, Japan; 4 Handa Health Center, 1-45-4 Deguchi-cho, Handa 475-0903, Japan; 5 Nishio Health Center, 12 Shimoda, Yorizumi-cho, Nishio 445-0073, Japan

**Keywords:** severe acute respiratory syndrome coronavirus 2, high-resolution melting, Omicron subvariant, BA.5, F486V mutation

## Abstract

Severe acute respiratory syndrome coronavirus 2 (SARS-CoV-2) Omicron subvariant BA.5 emerged as of February 2022 and replaced the earlier Omicron subvariants BA.1 and BA.2. COVID-19 genomic surveillance should be continued as new variants seem to subsequently appear, including post-BA.5 subvariants. A rapid assay is needed to differentiate between the currently dominant BA.5 variant and other variants. This study successfully developed a high-resolution melting (HRM)-based assay for BA.4/5-characteristic spike mutation F486V detection and demonstrated that our assay could discriminate between BA.1, BA.2, and BA.5 subvariants in clinical specimens. The mutational spectra at two regions (G446/L452 and F486) for the variant-selective HRM analysis was the focus of our assay. The mutational spectra used as the basis to identify each Omicron subvariant were as follows: BA.1 (G446S/L452/F486), BA.2 (G446/L452/F486), and BA.4/5 (G446/L452R/F486V). Upon mutation-coding RNA fragment analysis, the wild-type fragments melting curves were distinct from those of the mutant fragments. Based on the analysis of 120 clinical samples (40 each of subvariants BA.1, BA.2, and BA.5), this method’s sensitivity and specificity were determined to be more than 95% and 100%, respectively. These results clearly demonstrate that this HRM-based assay is a simple screening method for monitoring Omicron subvariant evolution.

## 1. Introduction

The coronavirus disease 2019 (COVID-19) outbreak was caused by the severe acute respiratory syndrome coronavirus 2 (SARS-CoV-2) that emerged in China in late 2019 [1,2,3]. The number of global COVID-19 cases surpassed 600 million according to the World Health Organization (WHO) data (Weekly epidemiological update on COVID-19, 7 September 2022), including over 6.4 million deaths. A number of SARS-CoV-2 variants emerged worldwide, some of which could potentially increase public health risk. The WHO has classified these as variants of concern (VOCs) and variants of interest (VOIs) since 2020. The COVID-19 genomic surveillance, tracking VOCs and VOIs, is essential in protecting against the spread of high-risk variants and monitoring new variants.

The novel VOC, the Omicron variant, emerged from South Africa in November 2021 [4,5,6]. The original Omicron variant BA.1 quickly spread to many countries and exceeded the other variants, including the Delta variant. The BA.2 subvariant of Omicron, which was more transmissible than BA.1 [7], rapidly increased since early 2022. The new Omicron subvariants BA.4 and BA.5 were first detected in South Africa in January and February 2022, respectively. The two subvariants subsequently became mainstream in the country. Although BA.4 did not spread further around the world, BA.5 quickly spread across the world after a few months from BA.2 becoming the dominant variant. Furthermore, close monitoring of the Omicron variants’ evolution should continue since additional Omicron subvariants seem to emerge subsequently [8].

Next-generation sequencing (NGS) is the gold standard for determining the whole viral genome. NGS data leads to assigning a lineage or clade to each SARS-CoV-2 strain. A screening assay, which identifies a SARS-CoV-2 point mutation, can contribute to the rapid identification of the individual variants within a day as NGS analysis requires a few days to acquire the data. A few previous studies have shown that screening assays can differentiate the Omicron subvariants BA.1 and BA.2 based on their characteristic mutations [9,10,11,12]. The BA.5 subvariant has two characteristic spike mutations, L452R and F486V (BA.4 sequence identical to BA.5 sequence in spike protein) [13,14]. Wilhelm et al. [15] reported that Omicron subvariants BA.4 and BA.5 in wastewater could be detected by commercially available kits for L452R and E484A/F486V. However, there are no or limited studies that demonstrate Omicron subvariant BA.5 identification in clinical specimens by a screening assay.

High-resolution melting (HRM) analysis is a post-PCR genotyping technique based on an amplicons’ melting behavior. HRM-based assay is a less-costly and quick-build method as the assay does not need a specific probe. Our HRM-based assay was previously demonstrated to discriminate between the Omicron BA.1 and BA.2 subvariants targeting three mutations in the spike receptor-binding domain (RBD), R408, G446/L452, and T477/T478 [16]. In this study, we aimed to (i) develop an HRM-based assay to detect the F486V mutation and (ii) validate the HRM-based assay for L452/G446 and F486 sites in 120 clinical samples (40 each of the BA.1, BA.2, and BA.5 subvariants). This HRM-based assay would contribute to rapid discernment between BA.1 (G446S, L452, and F486), BA.2 (G446, L452, and F486), and BA.4/5 (G446, L452R, and F486V) (Table 1).

## 2. Materials and Methods

### 2.1. Ethics Statement

The Meijo University Research Ethics Committee (Approval number: 2020-17-2) and Aichi Prefectural Institute of Public Health (Approval number: 20E-4) approved this project, and was carried out according to the Infectious Diseases Control Law of Japan.

### 2.2. Preparation of Standard RNA Fragments: In Vitro T7 Transcription

The SARS-CoV-2 sequence was obtained from the NCBI (GenBank ID: MN908947), the GISAID database (www.gisaid.org/; accessed on 9 August 2022), and the Pango nomenclature system (https://cov-lineages.org/lineages.html; accessed on 9 August 2022). Eurofins Genomics K.K provided the three RBD DNA fragments (wild-type, F486V mutant, BA.4/5 variant mutant; 600–1000 bp in length) with a 5′ T7 upstream promoter sequence. (Tokyo, Japan). In vitro T7 transcription was performed as previously described [16,17]. The synthesized single-stranded RNA fragments were used as reverse transcriptase (RT)-PCR amplification templates.

### 2.3. RT-PCR Amplification: First PCR

A one-step RT-PCR kit (One Step PrimeScript III RT-qPCR Mix, with UNG; TaKaRa Bio Inc., Kusatsu, Japan) was used to perform RT-PCR in a single closed tube in accordance with the manufacturer’s instructions. The primer pairs used for RT-PCR amplification are listed in Table 2. Each DNA fragment was observed as a single, correctly sized band (290 bp). RT-PCR amplification was performed as previously described [16,17]. The reaction mixture was diluted 10,000-fold with water and used as a template for the second PCR and HRM analyses after amplification.

### 2.4. HRM Analysis: Second PCR

**Figure 1:**
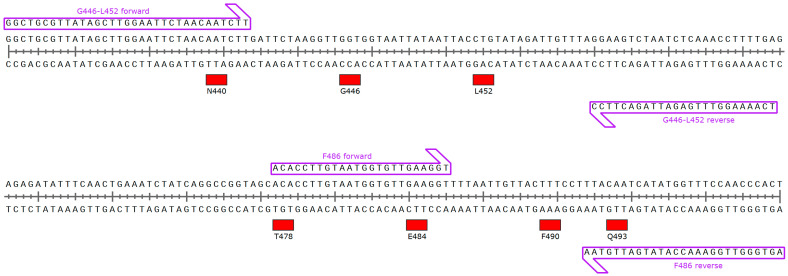
Primer annealing site schematic map for high-resolution melting analyses.

An HRM reagent (MeltDoctor HRM Master Mix; Thermo Fisher Scientific, Waltham, MA, USA) was used to perform HRM according to the manufacturer’s instructions. The primer pairs for HRM analysis are listed in Table 2. Each DNA fragment was observed as a single, correctly sized band (G466-L452, 104 bp; F486, 69 bp). The G446-L452 forward primer design was based on the N440 coding sequence, the F486 forward primer design was based on the T478 and E484 coding sequences, and the F486 reverse primer design was based on the Q493 coding sequence to avoid the potential influences of the N440, T478, E484, and Q493 mutations as shown in Figure 1. A real-time PCR system (LightCycler 96 System; Roche Diagnostics, Basel, Switzerland) was used to perform all reactions in duplicate. PCR amplification and HRM curves were performed as previously described [16,17].

### 2.5. Clinical Samples

From December 2021 to August 2022, 120 nasopharyngeal swabs or saliva samples were collected from suspected COVID-19 and those detected with COVID-19 by the Aichi Prefectural Institute of Public Health. The Ct values in the clinical quantitative PCR test for these samples ranged from 14–35. RNA purification and whole-genome sequencing were performed as previously described [16].

## 3. Results

### 3.1. Development of HRM Analysis for F486V Mutation Detection

**Figure 2:**
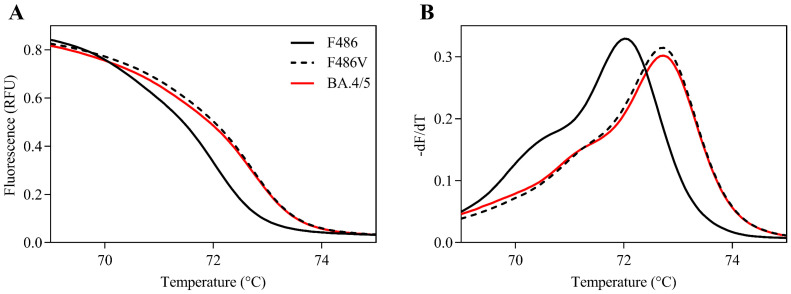
Positive control RNAs normalized melting curves and melting peaks for the F486 site. Normalized melting curve plots (**A**) and melting peak plots (**B**) for the F486 site were acquired using F486 receptor-binding domain standard fragments (RBD; solid black line), F486V RBD (dashed black line), and BA.4/5 RBD (solid red line).

First, the HRM-based assay for detecting F486V was developed, a characteristic mutation of BA.4/5. There are several SARS-CoV-2 variants possessing mutations near the F486 site, such as T478K, E484K, E484A, and Q493R. The risk of a false positive or false negative will likely increase in these variants using this assay. The primer sets were thus designed to avoid these mutations (Figure 1). The normalized melting curves and peaks for F486, F486V, and BA.4/5 RBD plots are shown in Figure 2. The F486V RBD plot was different from the F486 RBD plot. Additionally, the BA.4/5 RBD plot was in agreement with the F486V plot. These results suggested that this HRM-based assay can discriminate between the Omicron subvariant BA.4/5 (as the F486V variant) and other subvariants, including BA.1 and BA.2 (as the F486 wild-type).

### 3.2. Validation of HRM Analysis for the G446/L452 and F486 Sites in Clinical Samples

**Figure 3:**
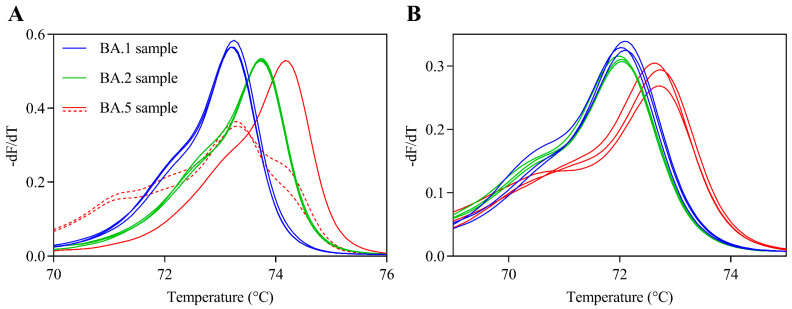
Melting peaks of clinical samples for two receptor-binding domain regions. Melting peak plots for the G446/L452 (**A**) and F486 (**B**) sites were acquired using clinical samples with three BA.1 samples (blue line), three BA.2 samples (green line), and three BA.5 samples (red line). Solid lines indicate true positive and true negative samples. Dashed lines indicate false positive samples.

Second, HRM-based assay was validated for G446/L452 and F486 sites in clinical samples infected with BA.1, BA.2, or BA.5. A total of 120 clinical samples (*n* = 40, BA.1; *n* = 40, BA.2; *n* = 40, BA.5) were randomly selected after whole-genome sequencing by NGS (Table 3). RBD sequences in all samples were identical to the reference sequence (BA.1, G446S/L452/F486; BA.2, G446/L452/F486; and BA.5, G446/L452R/F486V) confirmed by NGS data. HRM analysis together with positive control RNAs was used to analyze clinical samples. The Gene Scanning Software automatically classified samples into wild-type or mutant strains based on HRM curves after HRM analysis. The melting peak plots of representative samples at two RBD regions are shown in Figure 3. At the G446/L452 site, all BA.1 and BA.2 samples were correctly classified as G446S mutant and wild-type, respectively. The two BA.5 sample plots disagreed with the L452R RBD plot. All samples were correctly classified as each variant at the F486 site. Therefore, the sensitivity and specificity of the current HRM-based assay were calculated based on these results. The sensitivity and specificity of the assay for the two regions were more than 95% and 100%, respectively (Table 4).

## 4. Discussion

This is the first study conducted on developing a screening assay that can distinguish between the Omicron subvariant BA.5 and other subvariants in clinical samples. The assay costs less than USD 5 per sample, and the results can be obtained within 4 h. This HRM-based assay can be a powerful tool as a high-throughput screening test to identify the SARS-CoV-2 variants in many clinical samples.

The Omicron BA.5 subvariant seems to lead to lower mortality and hospitalization rates than earlier variants; however, the COVID-19 pandemic will not end this year [8]. A new high-risk variant will likely happen subsequently as the Omicron BA.5 subvariant is not considered the final SARS-CoV-2 variant [8]. Therefore, COVID-19 genomic surveillance should continue monitoring the next strain’s emergence. The BA.2.75 variant called Centaurus (non-WHO labeling) is one of the Omicron subvariants first detected in India [18,19,20,21]. BA.2.75’s potential infectivity and immune evasion are unclear, indicating that the variant should be closely monitored. The assay for N460S mutation detection was still developed as our HRM-based assay can be constructed quickly, which is a BA.2.75 subvariant characteristic mutation. We will continue developing HRM-based assays to detect new variants with potentially high public health risks.

This study needs to be interpreted in the context of its limitations. First, this assay cannot differentiate between BA.4 and BA.5 subvariants since both variants possess identical spike protein sequences (Table 1). The assay will be developed to identify the BA.4 specific mutations as necessary, such as OFR1a-DEL141/143, ORF7b-D61L, and N-P151S [22]. Second, this assay was validated using a limited sample number. Our HRM-based assay should analyze a larger sample size. Third, the current assay’s detection limit should be determined using low-copy virus samples with Ct values of more than 35.

## 5. Conclusions

We developed an HRM-based assay that detects the SARS-CoV-2 spike mutation F486V, known as the Omicron subvariants BA.4/5-characteristic mutation. Based on the analysis of clinical samples with BA.1, BA.2, and BA.5, our assay showed enough sensitivity and specificity to distinguish among the three subvariants. Further studies are needed to validate this HRM-based assay using diverse samples with Ct values of more than 35. However, the current HRM-based assay can construct a cost-effective screening test without any specific probe.

## Figures and Tables

**Table 1:** Receptor-binding domain (RBD) amino acid substitutions in Omicron BA.1, BA.2, BA.4, and BA.5 subvariants.

Pangolin	RBD Amino Acid Substitutions
BA.1	G339D, S371L, S373P, S375F, K417N, N440K, **G446S**, S477N, T478K, E484A, Q493R, G496S, Q498R, N501Y, Y505H
BA.2	G339D, S371F, S373P, S375F, T376A, D405N, R408S, K417N, N440K, S477N, T478K, E484A, Q493R, Q498R, N501Y, Y505H
BA.4/5	G339D, S371F, S373P, S375F, T376A, D405N, R408S, K417N, N440K, **L452R**, **F486V**, S477N, T478K, E484A, Q498R, N501Y, Y505H

Substitutions detected by this high-resolution melting assay are marked in bold.

**Table 2:** Primers sequences in reverse transcriptase (RT)-PCR amplification and high-resolution melting (HRM) analysis.

Primer	Sequence
RT-PCR amplification	
Fwd primer	5′-TTACAGGCTGCGTTATAG-3′
Rev primer	5′-ACAAACAGTTGCTGGTGCAT-3′
HRM analysis	
G446-L452 Fwd primer	5′-GGCTGCGTTATAGCTTGGAATTCTAACAATCTT-3′
G446-L452 Rev primer	5′-TCAAAAGGTTTGAGATTAGACTTCC-3′
F486 Fwd primer	5′-ACACCTTGTAATGGTGTTGAAGGT-3′
F486 Rev primer	5′-AGTGGGTTGGAAACCATATGATTGTAA-3′

Fwd: forward; Rev: reverse.

**Table 3:** Pangolin of 120 clinical samples by whole-genome sequencing.

BA.1			BA.2			BA.5		
Pangolin	No. of Samples	Ct Mean Value (Range)	Pangolin	No. of Samples	Ct Mean Value (Range)	Pangolin	No. of Samples	Ct Mean Value (Range)
BA.1	11	25.2 (22.2–29.9)	BA.2	10	23.0 (18.0–27.45)	BA.5	3	23.2 (16.9–27.9)
BA.1.1	5	24.2 (23.0–26.8)	BA.2.3	14	25.7 (20.1–34.9)	BA.5.1	2	22.2 (22.1–22.3)
BA.1.1.2	8	24.6 (20.0–29.9)	BA.2.3.1	7	23.9 (18.8–28.4)	BA.5.2	12	25.0 (19.2–29.1)
B.1.1.529	16	27.4 (21.1–32.6)	BA.2.3.13	1	20.8	BA.5.2.1	14	23.8 (14.3–29.1)
			BA.2.10	4	24.7 (22.9–28.7)	BA.5.3.1	1	22.1
			BA.2.10.2	1	23.6	BE.1.1	2	21.3 (17.9–24.8)
			BA.2.24	2	21.4 (18.4–24.5)	BF.5	6	24.1 (22.3–24.9)
			BA.2.29	1	28.8			

**Table 4:** High-resolution melting assay sensitivity and specificity compared with next-generation sequencing.

	G446S Detection	L452R Detection	F486V Detection
Sensitivity ^a^	100% (40/40)	95.0% (38/40)	100% (40/40)
Specificity ^b^	100% (80/80)	100% (80/80)	100% (80/80)

^a^ Sensitivity: True positive number/(True positive number + False Positive number) × 100. ^b^ Specificity: False negative number/(False negative number + True negative number) × 100.

## Data Availability

Data are unsuitable for public deposition due to ethical restrictions and the Infectious Diseases Control Law in Japan. The data presented in this study are available on request from the corresponding author. The data are not publicly available due to privacy restrictions.
